# Infection of (Peri-)Pancreatic Necrosis Is Associated with Increased Rates of Adverse Events during Endoscopic Drainage: A Retrospective Study

**DOI:** 10.3390/jcm11195851

**Published:** 2022-10-02

**Authors:** Fabian Frost, Laura Schlesinger, Mats L. Wiese, Steffi Urban, Sabrina von Rheinbaben, Quang Trung Tran, Christoph Budde, Markus M. Lerch, Tilman Pickartz, Ali A. Aghdassi

**Affiliations:** 1 Department of Medicine A, University Medicine Greifswald, 17475 Greifswald, Germany; 2 Department of Internal Medicine, University of Medicine and Pharmacy, Hue University, Hue 530000, Vietnam; 3 Ludwig Maximilian University Hospital, Ludwig Maximilian University of Munich, 81377 Munich, Germany

**Keywords:** stent, LAMS, WON, ANC, interventional EUS, pancreatitis

## Abstract

Pancreatic necroses are a major challenge in the treatment of patients with pancreatitis, causing high morbidity. When indicated, these lesions are usually drained endoscopically using plastic or metal stents. However, data on factors associated with the occurrence of failure or adverse events during stent therapy are scarce. We retrospectively analyzed all adverse events and their associated features which occurred in patients who underwent a first-time endoscopic drainage of pancreatic necrosis from 2009 to 2019. During the observation period, a total of 89 eligible cases were identified. Adverse events occurred in 58.4% of the cases, of which 76.9% were minor (e.g., stent dislocation, residual lesions, or stent obstruction). However, these events triggered repeated interventions (63.5% vs. 0%, *p* < 0.001) and prolonged hospital stays (21.0 [11.8–63.0] vs. 14.0 [7.0–31.0], *p* = 0.003) compared to controls without any adverse event. Important factors associated with the occurrence of adverse events during endoscopic drainage therapy were positive necrosis cultures (6.1 [2.3–16.1], OR [95% CI], *p* < 0.001) and a larger diameter of the treated lesion (1.3 [1.1–1.5], *p* < 0.001). Superinfection of pancreatic necrosis is the most significant factor increasing the likelihood of adverse events during endoscopic drainage. Therefore, control of infection is crucial for successful drainage therapy, and future studies need to consider superinfection of pancreatic necrosis as a possible confounding factor when comparing different therapeutic modalities.

## 1. Introduction

Acute pancreatitis is a non-malignant disease representing one of the most important gastrointestinal disorders leading to hospital admissions, with an increasing frequency over the past decades [1]. The most common triggers are excessive alcohol consumption and pancreatic duct obstruction by gallstones. Other risk factors include genetic, autoimmune, or metabolic diseases [2]. Pancreatic necroses are a feared complication in patients with pancreatitis as they lead to significant morbidity and mortality [3]. The Atlanta Classification [4] defines areas of necrosis as acute necrotic collections (ANC) until four weeks after the initial pancreatitis episode. Thereafter, these lesions usually develop a thickened wall, thus being named walled-off necrosis (WON). Drainage of pancreatic necrosis is indicated when complications occur such as infection, severe pain, or continuous enlargement of the lesion, causing obstruction of the gastric outlet or biliary obstruction [5]. Treatment of these lesions has changed dramatically within the last two decades, with an open surgical approach largely being replaced by endoscopic transmural drainage or minimally invasive surgical necrosectomy, as studies have shown significantly reduced mortality and high technical success rates with these methods [6]. Recent randomized controlled trials (RCTs) even showed that an endoscopic step-up approach resulted in lower rates of complications and shorter hospital stays when compared to a (minimally invasive) surgical step-up approach [7,8]. Other recently published RCTs [9,10] have found similar technical success rates when comparing an endoscopic with a laparoscopic drainage approach, while again, the endoscopic approach resulted in shorter hospital stays in one of the reports [10]. Therefore, endoscopic treatment currently represents the treatment modality of choice when drainage of pancreatic necrosis is indicated. The basic principle of endoscopic drainage is the creation of an orifice that connects the lesion with the gastrointestinal tract. Typically, a lumen-apposing metal stent (LAMS) or (multiple) plastic stent(s) is placed into the orifice to avoid its occlusion. This procedure also allows for repeated necrosectomies as needed. Typical challenges during endoscopic treatment can include stent dislocation, stent obstruction, systemic bacterial translocation, acute or delayed bleeding, residual lesions, or, in rare cases, intraabdominal perforations. The individual factors that contribute to the development of these adverse events are still not fully understood. In the present study, we retrospectively analyzed all cases where patients underwent a first-time endoscopic drainage of a pancreatic necrosis at a tertiary care hospital in the period from 2009 to 2019, analyzing the rates of adverse events during endoscopic drainage therapy and associated patient or treatment characteristics.

## 2. Materials and Methods

### 2.1. Study Participants and Phenotype Data

This retrospective single-center (University Medicine Greifswald) study analyzed all cases of first-time endoscopic drainage therapy of pancreatic necrosis in the period from January 2009 to December 2019. Potentially eligible cases were identified through a database search for endoscopic pancreatic drainage therapy in the hospital data administration system. The study was approved by the local institutional review board (registration no. BB 138-19).

Patients’ treatment data were extracted from medical records and laboratory charts. An age-adjusted Charlson Comorbidity Index [11] was calculated to summarize relevant comorbidities. Laboratory parameters were documented from the first 24 h of the patient’s admission. When any of the variables could not be obtained, they were considered as missing data. The location and diameter of the lesions were obtained from the available imaging data (computed tomography, magnetic resonance imaging, or endoscopic ultrasound) and radiologists’ reports. The duration of drainage therapy was determined from the stent placement until its extraction. Cases of (undocumented) spontaneous dislocation or loss to follow-up were considered as missing data. Pancreatic necroses were considered to be infected in cases where positive necrosis culture results were found. Single-shot antibiotics were also considered when determining the rate of antibiotic treatment. The variable ‘necessity for repeat interventions’ only summarized those interventions that were needed to treat adverse events and did not include regularly scheduled necrosectomies. Systemic inflammatory response syndrome (SIRS) after drainage was assigned if at least two SIRS criteria were positive [12]. Adverse events were considered minor in cases of simple stent dislocations; stent obstructions; residual lesions/unsuccessful drainage; SIRS after drainage responding to antimicrobial therapy within 72 h without necessity for intermediate or intensive care treatment; minor bleeding without shock or necessity of blood transfusion; or buried stent. Major complications were (intraabdominal) perforations, bleeding events with shock and/or necessity of blood transfusion, or any complication that resulted in intermediate or intensive care treatment. For determination of mortality rates, patients’ records were assessed up to six months after endoscopic drainage therapy or the patient’s last visit.

### 2.2. Endoscopic Drainage of Pancreatic Necrosis

In all cases, endoscopic ultrasound-guided transluminal (transgastric or transduodenal) drainage of pancreatic necrosis was performed. The complete sample comprised 84 WON and 5 ANC cases. All ANC cases had already developed a sufficiently matured wall at the time of drainage. Pancreatic necroses were identified using linear-array endoscopic ultrasound. For deployment of plastic (double) pigtail, Niti-S NAGI (TaeWoong Medical, Ilsan, Korea), or Niti-S SPAXUS stents (TaeWoong Medical), the lesions were punctured, a guidewire inserted, and a transluminal connection created using a cystotome. The stent was then inserted after balloon dilatation of the orifice. For implantation of Hot AXIOS stents (Boston Scientific, Marlborough, MA, USA), the pancreatic necroses were punctured using the electrocautery tip, the delivery catheter advanced into the collection, and the stent deployed. If necessary, the created orifice was used for repeated necrosectomies. Necrosectomies were performed on demand, taking into account the amount of necrotic material and its adhesion to the adjacent wall. Patients received either (multiple) plastic double pigtails or fully covered LAMS based on the endoscopist’s choice. Among the LAMSs, the Niti-S NAGI Stent (n = 30, TaeWoong Medical, Ilsan, Korea) and the Hot AXIOS (n = 26, Boston Scientific, Marlborough, MA, USA) stents were the most common choices (numbers include repeat interventions). A Niti-S SPAXUS Stent (TaeWoong Medical, Ilsan, Korea) was used on two occasions. In two cases, several different LAMSs were used consecutively (Hot AXIOS/Niti-S NAGI Stent/Niti-S SPAXUS Stent and Niti-S NAGI Stent/Hot AXIOS). In 14 cases, LAMSs and plastic pigtail stents were used consecutively. Generally, treatment was terminated and stents removed when the lesion disappeared or showed significant size reduction and when there were no signs of uncontrolled infection.

### 2.3. Data Analysis

All statistical analyses were performed using ‘R’ (v.3.6.3) [13]. For comparison of continuous data, a two-tailed *t*-test was employed (‘t.test’, ‘stats’ package), whereas categorical data were compared using the two-tailed Fisher’s exact test (‘fisher.test’, ‘stats’ package). Odds ratios (OR) and 95% confidence intervals (CI) were obtained from logistic regression models (‘glm’, family = ‘binomial’, ‘stats’ package) using the function ‘logistic.display’ (‘epiDisplay’ package) or ‘or_glm’ (‘oddsratio’ package) for categorical or continuous data, respectively. *p*-values < 0.05 were considered significant and rounded to three digits. Figures were created using the R package ‘ggplot2’.

## 3. Results

### 3.1. Rates and Types of Complications during Drainage Therapy

**Figure 1:**
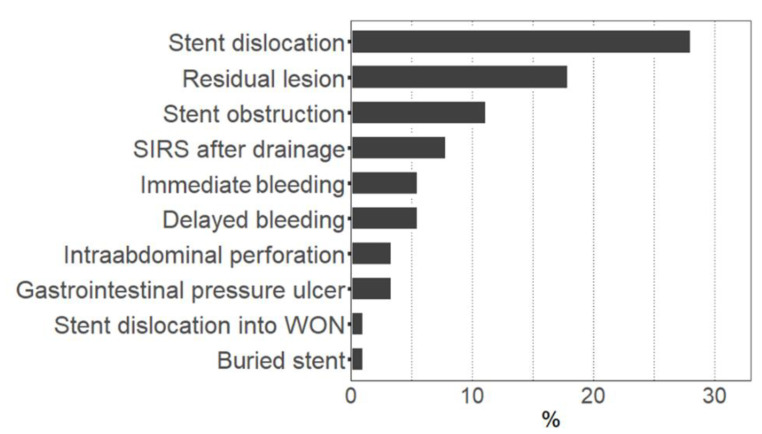
Rate of adverse events during endoscopic drainage therapy. Figure shows the percentage of cases with occurrence of the respective adverse event. Multiple adverse events in one case were possible. SIRS: Systemic inflammatory response syndrome. WON: Walled-off necrosis.

A total of 89 patients with pancreatic necrosis who underwent endoscopic drainage therapy were identified. Treatment-associated adverse events occurred in 52 cases (58.4%, ‘adverse events group’), whereas in 37 cases, no adverse events were observed (‘controls’). Minor adverse events represented the majority of these incidents (76.9%), and only 23.1% were major complications. The median time interval between drainage and the occurrence of any adverse event was 11 days (1–52.5 days, first–third quartile). The most common events were stent dislocation, residual lesion after drainage therapy, and stent obstruction (Figure 1). Other observed adverse events included occurrence of SIRS after drainage, immediate or delayed bleedings, intraabdominal perforation during stent placement, stent-induced gastrointestinal pressure ulcers, or buried stent syndrome. Patients’ baseline characteristics were very similar in the adverse events group when compared to controls (Table 1). No difference could be found with regard to age, sex, body mass index, type and etiology of pancreatitis, severity of comorbidities (as indicated by age-adjusted Charlson Comorbidity Index), rate of pancreatic enzyme replacement therapy, or different laboratory parameters. The only significant difference was a higher proportion of diabetics in the control group.

### 3.2. Treatment Characteristics of Adverse Events Cases and Controls

**Figure 2:**
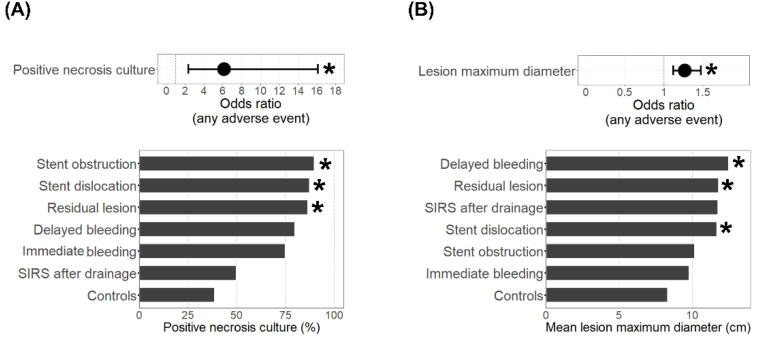
Treatment characteristics of adverse events cases. Shown are the odds ratios (95% confidence interval) for the occurrence of any adverse event (**top**) and the rates of positive necrosis cultures (**A**) or the mean lesion maximum diameter (**B**) in cases with the respective adverse event (**bottom**). * Indicates a significant (*p* < 0.05) difference compared to the controls.

When comparing the treatment characteristics between the adverse events group and controls (Table 2), we found infection of pancreatic necrosis (6.1 [2.3–16.1], OR [95% CI], *p* < 0.001) as indicated by a positive culture, as well as a higher lesion maximum diameter (1.3 [1.1–1.5], OR [95% CI], increment 1 cm, *p* < 0.001), to be associated with adverse events during endoscopic drainage therapy (Figure 2). More specifically, a lesion diameter > 10 cm was linked to an OR of 4.6 (1.8–11.9; 95% CI, *p* = 0.001) for the occurrence of any adverse event. When including a positive culture and lesion maximum diameter in one model using age, sex, and diabetes mellitus as covariates, a positive necrosis culture (*p* = 0.002), as well as a maximum diameter (*p* = 0.001), remained significantly associated with the occurrence of adverse events during endoscopic drainage therapy. The presence of bacteria in areas of pancreatic necrosis showed the strongest association with adverse events such as stent dislocation (*p* < 0.001), residual lesion (*p* = 0.002), and stent obstruction (*p* = 0.010) (Figure 2A). In cases with immediate or delayed bleeding incidents, 77.8% showed pancreatic necrosis infections, but that rate was not significantly (*p* = 0.061) higher compared to 38.9% in the controls. A larger maximum diameter of the lesion was associated with delayed bleeding (*p* = 0.007), residual lesion (*p* = 0.004), or stent dislocation (*p* = 0.002) (Figure 2B). The adverse events group’s initial hospital stays also had a longer duration (*p* = 0.003). No significant difference could be found with respect to the type of stent being used (plastic or LAMS, Table 3). A repeat intervention was necessary in 63.5% of the cases with adverse events and was performed endoscopically in the majority of cases. Additional radiological percutaneous drainage or intervention was needed in 28.8% of all cases, and a surgical approach in 9.6% of all cases. The mortality was higher in the adverse events group when compared to the controls (15.4% vs. 5.4%); however, this was not significant. One patient in the adverse events group died due to a fatal delayed bleeding incident 11 days after LAMS placement. Apart from this single case, there was no treatment-associated mortality observed in this cohort.

## 4. Discussion

We analyzed patient and treatment characteristics of cases with adverse events during endoscopic transluminal drainage therapy of pancreatic necrosis as compared to controls without adverse events. Although the overall rate of adverse events was high (58.4%), most of them (76.9%) were minor and could be treated endoscopically and/or by radiological intervention. Patients’ baseline characteristics were very similar in both groups, with no differences in age, sex, body mass index, etiology of pancreatitis, or severity of comorbidities. However, the most prominent factor associated with adverse events during endoscopic drainage therapy was positive necrosis cultures indicating superinfection. It is believed that infection of pancreatic necrosis occurs via translocation of commensal gut bacteria. The (healthy) exocrine pancreas plays an important role for gut microbiome regulation [14,15]. A combination of intestinal dysbiosis in patients with pancreatitis [16,17], local and systemic immunosuppression, and a disturbed barrier function can promote the translocation of gut bacteria into areas of necrosis, as has been shown in a rodent model [18]. Consequently, the microorganisms identified in pancreatic necroses belonged largely to the intestinal gut flora (e.g., *Enterococcus faecium*, *Candida albicans,* or *Escherichia (E.) coli*, Figures S1 and S2) and were similar to those previously identified in other studies [19,20]. Apparently, the presence of these microorganisms has a negative effect on the success of the endoscopic drainage therapy. Cases with stent obstruction, stent dislocation, or residual lesions after drainage showed especially high rates of positive culture results. Cases with bleeding incidents showed higher rates of pancreatic necrosis infections compared to controls (77.8% vs. 38.9%); however, this was not significant. Higher rates of stent obstruction could be explained by microbial overgrowth of the stent surface by agglutinative bacteria and microbial biofilm development, as has already been shown for obstructed biliary stents [21]. A range of Gram-negative or Gram-positive bacteria or yeast such as *C. albicans* possess the capability for biofilm development and agglutination [22,23]. Similarly, biofilm development may impair drainage of pancreatic necrosis in the cavity itself, leading to higher rates of residual lesions. Stent dislocation, on the other hand, could be the result of microbe-induced inflammation, impairing wound healing and loosening the stent fixation in the cavity. In an in vitro model, *E. coli*-derived cytotoxic necrotizing factor type 1 impaired intestinal epithelial wound repair after an experimental mechanical trauma [24]. The bacterial endotoxin lipopolysaccharide (LPS), which is the major component of the outer membrane of Gram-negative bacteria such as *E. coli*, leads to impaired blood flow and a proinflammatory immune response, which resulted in insufficient healing of gastric ulcers in the rat model [25]. Other bacteria that are frequently found in necrosis isolates such as *Enterococcus faecium*, *Staphylococcus aureus*, and *Pseudomonas aeruginosa*, or LPS more generally, have also been shown in vitro and/or in vivo to secrete compounds that interfere with the host’s immune response or epithelial cell migration and may, therefore, impair wound healing [26,27]. Higher rates of (delayed) bleeding events in cases with pancreatic necrosis infection may similarly result from increased inflammation in consecutively impaired wound healing and increased erosion of blood vessels.

As a second factor associated with adverse events during endoscopic drainage therapy, we identified a larger pancreatic necrosis diameter. This likely reflects the consequence of more severe states of the disease and increased technical difficulty in stent placement due to the anatomical position.

Although the basic phenotype characteristics were very similar between the adverse events group and controls, the latter included a larger proportion of diabetics. An explanation could be the typically increased rates of reduced exocrine pancreatic function [28] in diabetics, possibly resulting in reduced (auto-)proteolytic activity and inflammation during pancreatitis within the area of necrosis, leading to less adverse events. Of note, adding diabetes mellitus as a covariate to the regression model had no relevant impact on the significance of the association of infected pancreatic necrosis or lesion maximum diameter with the occurrence of adverse events.

There is still ongoing debate on the (non-)superiority of LAMSs compared to plastic stents in the drainage of pancreatic necrosis. A retrospective study [29] observed higher rates of residual lesions after drainage therapy with plastic stents. Likewise, in the present study, residual lesions were observed in 22.9% of cases when plastic stents were used, but only in 10.9% when LAMSs were used. However, this difference was not significant. Stent obstruction was (again not significantly) more common when LAMSs were used, but endoscopists may have underreported stent obstruction when (thinner) plastic stents were used as it is less easy to detect. The only fatal therapy-associated complication was a major delayed erosion bleed 11 days after LAMS placement, which underlines the risk of rare severe bleeding events when LAMSs are being used [30]. Regarding other adverse events, no apparent difference between LAMS and plastic stent usage could be detected. Retrospective studies have the inherent limitation that the initial presentation of the collection may bias the endoscopist’s choice between a plastic stent or LAMS, which influences the outcome. In one randomized controlled trial (RCT) that included 31 WON patients with LAMS and 29 with plastic stent treatment, no significant difference (except for the duration of the procedure) could be found [31]. This is further supported by a recent meta-analysis that found no difference in the occurrence of adverse events between LAMSs and plastic stents in the treatment of WON when only including studies with EUS-guided drainage [32]. Further RCTs rather, than retrospective studies, are needed to investigate whether newly developed LAMSs or plastic stents are superior, or whether the two stent types’ treatment quality is equal. Moreover, the performance of the different stent types may also depend on the lesion’s composition. The choice of LAMS or plastic stents could be made according to the amount of necrotic debris in the target lesion, with LAMS used only for lesions with a large proportion of solid components. This approach has been applied in a recently published RCT [10], achieving similar success rates compared to a laparoscopic drainage approach.

Despite the thorough retrospective analysis, this study has some limitations. First, as this is a single-center study, the total sample size is limited, and in particular, smaller differences between the groups associated with rarer adverse events could have escaped detection. Second, the usage of culture results to determine infection of pancreatic necrosis has its limitations as diverse microbial communities and anaerobic bacteria cannot be reliably detected. This would require the usage of next-generation sequencing techniques, which are still not a part of the clinical routine.

To summarize, our data show that infection of pancreatic necrosis is the most significant factor associated with adverse events during endoscopic transluminal drainage therapy. Apart from optimizing diagnosis and treatment of the infection itself, the data indicate a potential to optimize stents for deployment in areas of infection, e.g., by using antimicrobial coatings. Similar approaches are currently under development for stent therapy in the biliary tract [33,34]. Whether such an approach can be translated into the treatment of pancreatic necrosis, however, needs to be investigated in further experimental studies. Moreover, future studies that aim to compare different methods of endoscopic drainage or stent therapy need to consider infection with pancreatic necrosis as a possible confounding factor.

## Figures and Tables

**Table 1:** Baseline phenotype characteristics of cases with adverse events and uncomplicated controls.

	Adverse Events Group (n = 52)	No Complication/ Controls (n = 37)	Missing (%)	*p*-Value
Age (years)	62.5 (46.8–69.2)	55.0 (48.0–63.0)	0	0.292
Female sex (%)	26.9	24.3	0	0.811
Body mass index (kg/m²)	24.5 (22.5–26.9)	24.3 (22.3–26.7)	7.5	0.693
Smoking history	56.1	66.7	13.5	0.361
Etiology of pancreatitis (%)			0	0.763
Alcoholic	40.4	48.6		
Biliary	34.6	24.3		
Idiopathic	19.2	21.6		
Other	5.8	5.4		
Charlson Comorbidity Index	3.0 (2.0–5.0)	3.0 (1.0–5.0)	0	0.441
Diabetes mellitus (%)	19.2	40.5	0	0.033 *
PERT (%)	28.8	40.5	0	0.265
History of non-pancreatic malignancy (%)	7.7	5.4	0	1.000
History of abdominal surgery (%)	21.2	27.0	0	0.615
White blood cells (Gpt/L)	11.4 (8.2–18.1)	10.5 (9.0–16.0)	0	0.784
Hemoglobin (mmol/L)	7.6 (6.2–8.6)	7.5 (6.6–8.2)	0	0.916
Hematocrit (%)	37.6 (31.8–43.2)	37.0 (33.6–40.2)	0	0.994
Platelet count (Gpt/L)	278.5 (208.2–389.5)	303.0 (197.0–384.0)	0	0.746
eGFR < 60 mL/min (%)	28.8	16.2	0	0.210
Blood urea nitrogen (mmol/L)	2.3 (1.8–4.2)	2.3 (1.6–3.2)	4.5	0.168
Albumin (g/L)	26.0 (20.0–35.0)	28.0 (23.2–33.2)	18.0	0.475
Lipase (µkatal/L)	7.4 (2.7–68.2)	7.2 (2.1–16.2)	9.0	0.104
ALT (µkatal/L)	0.5 (0.3–0.8)	0.4 (0.3–0.6)	4.5	0.130
Bilirubin (µmol/L)	8.5 (5.7–13.1)	8.7 (5.7–11.4)	0	0.494
CRP (mg/L)	111.5 (10.0–196.8)	114.0 (13.8–210.8)	1.1	0.878

Continuous data are given as median (first–third quartile). Categorical variables are displayed as percentages. All values are rounded to one decimal place. * Indicates significant result (*p* < 0.05). ALT: Alanine aminotransferase. CRP: C-reactive protein. eGFR: Estimated glomerular filtration rate. n: Number of cases. PERT: Pancreatic enzyme replacement therapy.

**Table 2:** Treatment characteristics of cases with adverse events and uncomplicated controls.

	Adverse Events Group (n = 52)	No Complication/ Controls (n = 37)	Missing (%)	*p*-Value
Indication for drainage (%, multiple possible)				
Suspected infection	51.9	48.6	0	0.831
Pain (only)	19.2	18.9	0	1.000
Continuous enlargement of lesion	13.5	16.2	0	0.767
Gastric outlet obstruction	11.5	13.5	0	1.000
Biliary obstruction	1.9	5.4	0	0.568
Other	11.5	2.7	0	0.232
Type of stent used for initial treatment (%)			0	0.384
Plastic pigtail stent(s)	46.2	35.1		
LAMS	53.8	64.9		
Type of lesion (%)				0.645
WON	96.2	91.9	0	
ANC	3.8	8.1	0	
Location of lesion (%, multiple possible)				
Head	30.8	27.0	0	0.814
Body	59.6	45.9	0	0.281
Tail	50.0	59.5	0	0.398
Lesion maximum diameter (cm)	10.9 (8.4–15.1)	7.6 (6.0–10.0)	0	<0.001 *
Necrosis culture: positive results (%)	79.6	38.9	4.5	<0.001 *
Blood culture: positive results (%)	26.3	21.1	36.0	0.754
Antibiotic treatment (%)	100.0	97.3	0	0.416
Highest level of care (%)				0.054
Intensive care unit	48.1	27.0	0	
Intermediate care	26.9	24.3	0	
Regular ward	25.0	48.6	0	
Endoscopic necrosectomy performed (%)	53.9	48.7	0	0.671
Interval (days) between initial drainage and first necrosectomy	6.5 (3.8–11.0)	3.5 (2.2–5.0)	0	0.077
Necessity for repeat interventions (%, multiple possible)	63.5	0	0	<0.001 *
Endoscopic	44.2	-		
Interventional radiology	28.8	-		
Surgical	9.6	-		
Duration of initial hospital stay (days)	21.0 (11.8–63.0)	14.0 (7.0–31.0)	0	0.003 *
Duration of endoscopic drainage (days)	65.0 (47.8–103.2)	64.5 (51.2–129.0)	9.5	0.853
Total mortality (%)	15.4	5.4	0	0.185
Therapy-related mortality (%)	1.9	0	0	1.000

Continuous data are given as the median (first–third quartile). Categorical variables are displayed as percentages. All values are rounded to one decimal place. * Indicates a significant result (*p* < 0.05). ANC: Acute necrotic collection. n: Number of cases. LAMS: Lumen-apposing metal stent. WON: Walled-off necrosis.

**Table 3:** Comparison of adverse events frequency between lumen-apposing metal stent (LAMS) and plastic stent usage.

	LAMS (n = 55)	Plastic Stents (n = 48)	*p*-Value
Stent dislocation	21.8	29.2	0.496
Residual lesion	10.9	22.9	0.118
Stent obstruction	12.7	4.2	0.170
SIRS after drainage	3.6	10.4	0.247
Immediate bleeding	3.6	4.2	1.000
Delayed bleeding	7.3	2.1	0.369
Other rare complications	10.9	6.2	0.498
Complication-associated fatality	1.8	0	1.000
Any adverse event	49.1	54.2	0.694

Categorical variables are displayed as percentages. All values are rounded to one decimal place. n: Number of cases. SIRS: Systemic inflammatory response syndrome.

## Data Availability

Data are available from the corresponding author on reasonable request.

## Supplementary Materials

The following supporting information can be downloaded at: https://www.mdpi.com/article/10.3390/jcm11195851/s1, Figure S1: Necrosis culture results, Figure S2: Comparison of necrosis culture results between controls and adverse events cases.
