# Supplementary material for: Infection of (Peri-)Pancreatic Necrosis Is Associated with Increased Rates of Adverse Events during Endoscopic Drainage: A Retrospective Study
Source: J Clin Med. 2022 Oct 2;11(19):5851. doi: 10.3390/jcm11195851 (PMC9573742; doi:10.3390/jcm11195851)
Supplement: Supplementary file 1 [file jcm-11-05851-s001.zip › jcm-1891001-supplementary.pdf]

Infection of (Peri-)Pancreatic Necrosis Is Associated with Increased Rates of Adverse Events during Endoscopic Drainage: A Retrospective Study

Fabian Frost [1,*], Laura Schlesinger [1], Mats L. Wiese [1], Steffi Urban [1], Sabrina von Rheinbaben [1], Quang Trung Tran [1,2], Christoph Budde [1], Markus M. Lerch [1,3], Tilman Pickartz [1,†] and Ali A. Aghdassi [1,†]

[1] Department of Medicine A, University Medicine Greifswald, 17475 Greifswald, Germany

[2] Department of Internal Medicine, University of Medicine and Pharmacy, Hue University, Hue 530000, Vietnam

[3] Ludwig Maximilian University Hospital, Ludwig Maximilian University of Munich, 81377 Munich, Germany

* Correspondence: fabian.frost@med.uni-greifswald.de; Tel.: +49-3834-86-7230

† These authors contributed equally to this work.

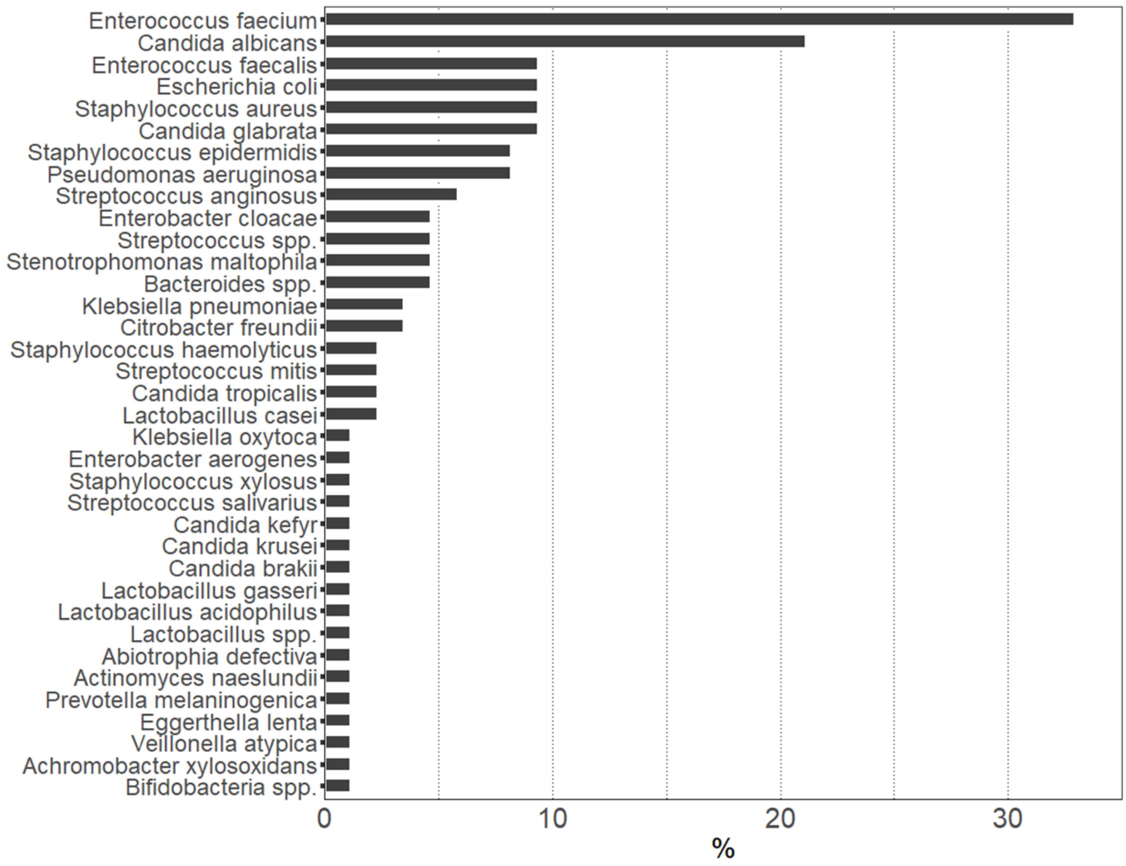

Figure S1. Necrosis culture results. Shown are the rates of positive necrosis cultures for the respective bacterial or fungal species.

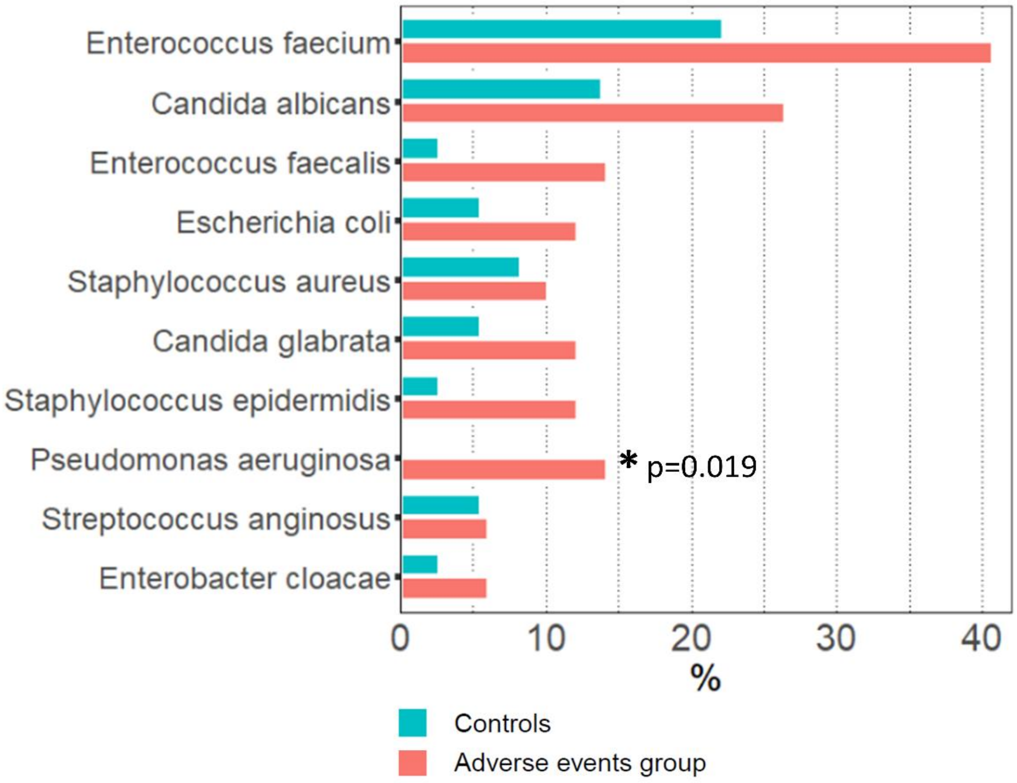

Figure S2. Comparison of necrosis culture results between controls and adverse events cases. Shown are the eight most frequently found taxa. * indicates significant ($p<0.05$) difference.
